# Vitamin and Mineral Deficiency 12 Years After Roux-en-Y Gastric Bypass a Cross-Sectional Multicenter Study

**DOI:** 10.1007/s11695-023-06787-w

**Published:** 2023-08-27

**Authors:** Kirsti K. Bjerkan, Jorunn Sandvik, Siren Nymo, Halvor Græslie, Gjermund Johnsen, Ronald Mårvik, Åsne A. Hyldmo, Bård Eirik Kulseng, Sandra Sommerseth, Kjetil Laurits Høydal, Dag Arne L. Hoff

**Affiliations:** 1 Faculty of Social Science and History, Volda University College, Joplassvegen 1, Volda, 6103 Norway; 2 Department of Surgery, Møre and Romsdal Hospital Trust, Ålesund, Norway; 3 Centre for Obesity Research, Clinic of Surgery, St. Olav’s University Hospital, Trondheim, Norway; 4 Department of Clinical and Molecular Medicine, Faculty of Medicine and Health Sciences, Norwegian University of Science and Technology, Trondheim, Norway; 5 Clinic of Surgery, Namsos Hospital, Nord-Trøndelag Hospital Trust, Namsos, Norway; 6 Norwegian National Advisory Unit On Advanced Laparoscopic Surgery, Clinic of Surgery, St. Olav’s University Hospital, Trondheim, Norway; 7 Department of Neuromedicine and Movement Science, Faculty and Medicine and Health Sciences, NTNU, Trondheim, Norway; 8 Department of Physical Education, Faculty of Arts and Physical Education, Volda University College, Volda, Norway; 9 Department of Research and Innovation, Møre and Romsdal Hospital Trust, Ålesund, Norway

**Keywords:** Gastric Bypass, Vitamin and mineral deficiency, Long-term follow-up, Vitamin D, Vitamin B_12_

## Abstract

**Purpose:**

Micronutrient deficiencies are common after Roux-en-Y gastric bypass (RYGB). This study explores whether vitamin and mineral deficiency was associated with adherence to recommended supplementation 12 years after RYGB.

**Materials and Methods:**

The cross-sectional Bariatric Surgery Observation Study (BAROBS) was conducted in 2018–2020 at three hospitals in Central Norway. We report data on 490 patients’ self-reported adherence to recommended supplements and vitamin and mineral levels in the blood. The patients, who had RYGB between 2003 and 2009, were recommended an over-the-counter multivitamin-mineral supplement, calcium/vitamin D (1000 mg/20 µg) and vitamin B_12_ injections (reimbursed), since bariatric supplements were not available then.

**Results:**

Mean (SD) age was 40.1 ± 9 years at RYGB, and time to follow-up 11.7 ± 1.6 years. Of 490 patients, 393 (80%) were women. Among 361 (74%) patients’ adherent to multivitamin-mineral supplements; folate, vitamin B_2,_ and vitamin B_6_ deficiency were present in 39 (11%), 103 (29%), and 63 (17%) patients, respectively. The same deficiencies occurred in 44 (34%), 67 (52%), and 67 (52%) patients’ non-adherent to recommendations. Although 466 (95%) patients reported adherence to vitamin B_12_ supplements, sub-optimal levels were found in 73 (16%) patients.

Though 336 (69%) patients adhered to calcium/vitamin D supplements (1000 mg/20 µg), sub-optimal vitamin D levels (< 75 nmol/l) were found in 174/336 (52%) adherent patients and 120/154 (78%) non-adherent patients.

**Conclusion:**

Twelve years after RYGB, adherence to supplements, though in sub-optimal doses of new recommendations, decreases the probability of vitamin and mineral deficiency, especially for thiamine, vitamin B_2_, vitamin B_6_, folate, vitamin B_12_, and vitamin D, but does not eliminate it.

**Graphical Abstract:**

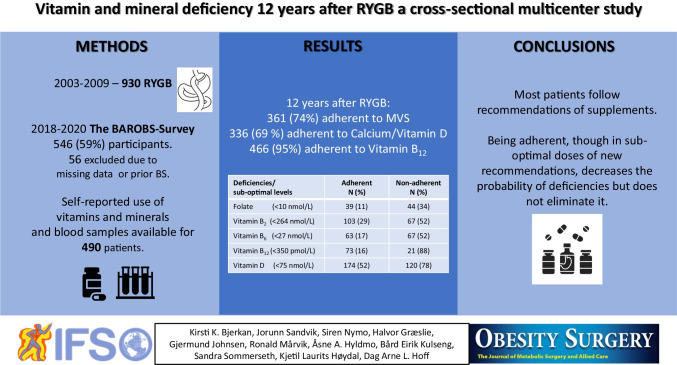

**Supplementary Information:**

The online version contains supplementary material available at 10.1007/s11695-023-06787-w.

## Introduction

### Background

Roux-en-Y gastric bypass (RYGB) for treating severe obesity leads to reduced uptake of vitamins and minerals from food, as the duodenum, the most important area for absorption of micronutrients, is bypassed [1, 2]. The most commonly reported nutritional deficiencies after bariatric surgery are iron, folate, vitamin B_12_, copper, zinc, calcium, and vitamin D [1, 3]. The guidelines for supplementation after bariatric surgery aim to give simple recommendations which should be easy and affordable for all patients and give a reasonable balance between benefits and side effects [4, 5]. Former studies have found prevalence of micronutrient deficiency in patients without supplementation after obesity surgery that ranges from 15 to 38% in folic acid, 37–50% in vitamin B_12_ and 47–66% for iron and vitamin D from 20 to 80% related to geographical areas [6]. A Norwegian study from 2015 found that 70% of the patients used multivitamin, 52% used calcium with vitamin D and 83% used vitamin B_12_ injections after 5 years. In addition, a decrease in adherence rate from 1 to 5 years after surgery for all supplements except for vitamin B_12_ was found [7].

Younger patients, patients with major comorbidities, low socioeconomic status, surgical complications, and those with great weight loss, the first year after surgery are more prone to vitamin and mineral deficiency [3, 6]. Several studies have documented that adherence to recommended supplements declines over time after the surgical procedure. Termination due to lack of education may influence, as well as taste and price [6, 8–11].

### Objective

This study aims to explore whether patients followed the recommendations on life-long supplementation of vitamins and minerals more than a decade after RYGB surgery and whether patients adhering to supplements had fewer deficiencies compared to patients not following the recommendations.

## Methods

### Study Design

**Fig. 1:**
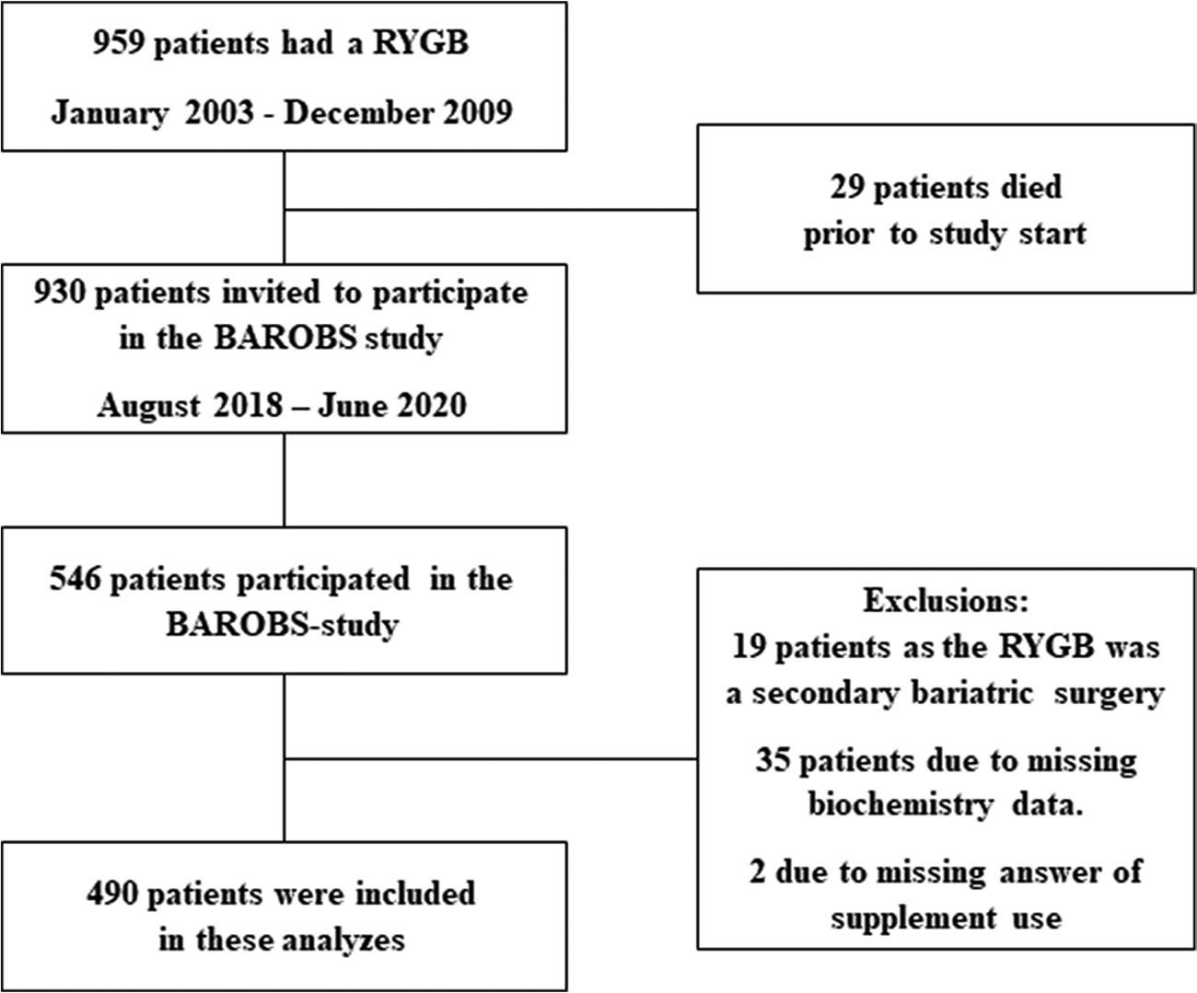
The recruitment procedure in the Bariatric Surgery Observation Study (BAROBS) of patients operated by the Roux-en-Y gastric bypass (RYGB) surgical method

The Bariatric Surgery Observation Study (BAROBS) is a cross-sectional observation study including all patients who underwent RYGB from 2003 to 2009 in the Central Norway Health Region to a follow-up study from 2018 to 2020. Of 930 invited patients, 546 (59%) participated. Fifty-six patients were excluded from the analyses, presented in the flowchart (Fig. 1). Data from 490 patients on self-reported vitamin and mineral intake and vitamin/mineral status in blood samples have been analyzed. All patients had postoperatively been followed at the outpatient clinic for 2 to 5 years and were invited to participate in an educational program lasting 2 to 3 years [12]. The patients were recommended yearly blood test of ferritin, iron, vitamin D, folate, and vitamin B_12_. The RYGB procedure in these patients was standardized and performed laparoscopically according to the Lönroth technique; a pouch size of 30–50 ml and with an ante-gastric alimentary limb of 100 cm or 150 cm, depending on BMI below or above 50 kg/m^2^, and a biliopancreatic limb of approximately 50 cm [13].

### Postoperative Vitamin and Mineral Recommendations

At time of operation 2003–2009, all patients were recommended life-long supplementation of vitamins and minerals, including an over-the-counter multivitamin-mineral supplement, 1000 mg of calcium carbonate including 20 µg vitamin D, and Vitamin B_12_ given as an injection of hydroxocobalamin or per oral cyanocobalamin. Only the B_12_ was covered by reimbursement, which leaves the multivitamin-mineral and the calcium/vitamin D supplement to be paid by the patients. The most frequently used multivitamin-mineral supplement is shown in Table 1. To avoid iron deficiency, per oral iron supplementation was recommended either continuously or intermittently based on ferritin levels at an annual follow-up. Data on iron deficiency have been published in a separate paper [14].

**Table 1:** Content of vitamins, minerals and trace elements in the most frequently used multivitamin-mineral supplement

Vitamins/minerals/trace elements	Dose
Vitamin A, µg RAE	250
Vitamin B1 (thiamine), mg	1.4
Vitamin B2, (riboflavin) mg	1.6
Vitamin B3 (niacin), mg NE	19
Vitamin B5 (pantothenic acid), mg	5
Vitamin B6 (pyridoxine), mg	1.6
Biotin, µg	30
Folic acid, µg	400
Vitamin B12, µg	2
Vitamin C, mg	75
Vitamin D, µg (IE)	10 (400)
Vitamin E, mg TE	10
Vitamin K1, µg	120
Chrome, µg	35
Iron, mg	15
Iodine, µg	150
Copper, µg	900
Magnesium, mg	100
Manganese, mg	2.3
Molybdenum, µg	45
Selenium, µg	60
Zink, mg	12
Calcium, mg	0

### Measurements

Adherence to supplements was self-reported. Fasting venous blood samples were analyzed for thiamine (vitamin B_1_), vitamin B_2_, vitamin B_6_, folate, vitamin B_12_, vitamin A, vitamin D (measured as the sum of vitamin D_2_ (25-OH-kalciferol), and vitamin D_3_ (25-OH-kalcidiol)), free calcium, parathyroid hormone (PTH), zinc, copper, methylmalonic acid (MMA), and homocysteine. Samples not analyzed locally were frozen at − 20 degrees Celsius and sent to a collaborating laboratory for analysis. This is applied to zinc, copper, vitamin A, vitamin B_1_, vitamin B_2_, and vitamin B_6_. We use the decision level of 10 nmol/L for folate according to the recommendation from WHO, 75 nmol/L for vitamin D and 350 pmol/L for vitamin B_12_ and the lower reference value of 186 pmol/L [4, 15–17]. All the laboratories in the study were certified according to ISO:15189. The instruments used for analysis are listed in Supplement 1. PTH is reported as within or above the reference range as the hospitals used different methods and reference values.

Patients are defined to be adherent or non-adherent in three categories: multivitamin-mineral supplements, vitamin B_12_, and/or calcium with vitamin D.

### Statistics

Categorical variables are presented as frequency and percentages and compared using Pearson *χ*2, *p*-values. Normally distributed data are presented as mean ± SD while non-normally distributed data as the median and interquartile range (Q1–Q3). To compare normally and non-normally distributed data, independent *t*-test and Mann–Whitney were used, respectively. A two-way ANOVA was used to estimate how the mean of quantitative variables changes according to the levels of two categorical variables. Binary logistic regression was performed to find the odds ratio (OR) for nutritional deficiencies in adherent vs non-adherent patients. *p*-values < 0.05 was considered significant. We used IBM SPSS statistics version 29 (SPSS Inc., Chicago, IL, USA) and GraphPad Prism version 9 (GraphPad Software, LLC, CA, USA).

## Results

The 490 patients had a mean ± SD age at surgery of 40.1 ± 9.0 years and 51.8 ± 9.0 years at follow-up, after 11.7 ± 1.6 years. Three-hundred and ninety-three (80%) were women. Body mass index (BMI) changed from 44.4 ± 5.4 kg/m^2^ to 35.0 ± 7.0 kg/m^2^ (Table 2). A total of 361 (74%) patients reported being adherent to multivitamin-mineral supplements at follow-up, adherence to vitamin B_12_ was 466 (95%), and calcium with vitamin D was 336 (69%). Among adherent patients, 319/361 (83%) used the same multivitamin-mineral supplement (see Table 1). Per oral vitamin B_12_ was used in 110/466 (25%) of the adherent patients, while 356/466 (75%) used hydroxocobalamin injections. The recommended calcium with vitamin D (1000 mg/20 µg) was used in 305/336 (91%) adherent patients.

**Table 2:** General characteristics of patient’s (n) adherent (A) or non-adherent (NA) to lifelong multivitamin-mineral supplementation

*n* = 490	A *n* = 361 (74%)	NA *n* = 129 (26%)	*p*-value
Sex W/M	295/66	98/31	
Age at baseline (SD) kg/m^2^	40.4 (9.0)	39.4 (8.9)	ns
Mean follow-up time (SD) months	140.1 (19.4)	142.1 (18.0)	ns
BMI at baseline (SD) kg/m^2^	44.2 (5.3)	44.9 (5.6)	ns
BMI at 10–15 years (SD) kg/m^2^	34.5 (6.8)	36.4 (7.3)	0.008
Level of education n (%) < 12 year of school > 12 year of school	226/347 (65) 121/347 (35)	92/127 (72) 35/127 (28)	*p* = 0.134*
Level of income n (%) < 75,000 Euro > 75,000 Euro	220/346 (64) 126/346 (36)	93/127 (73) 34/127 (27)	*p* = 0.049*

*W* women, *M* = men

*SD* Standard deviation

^*^Data analyzed with Pearson Chi-Square

**Fig. 2:**
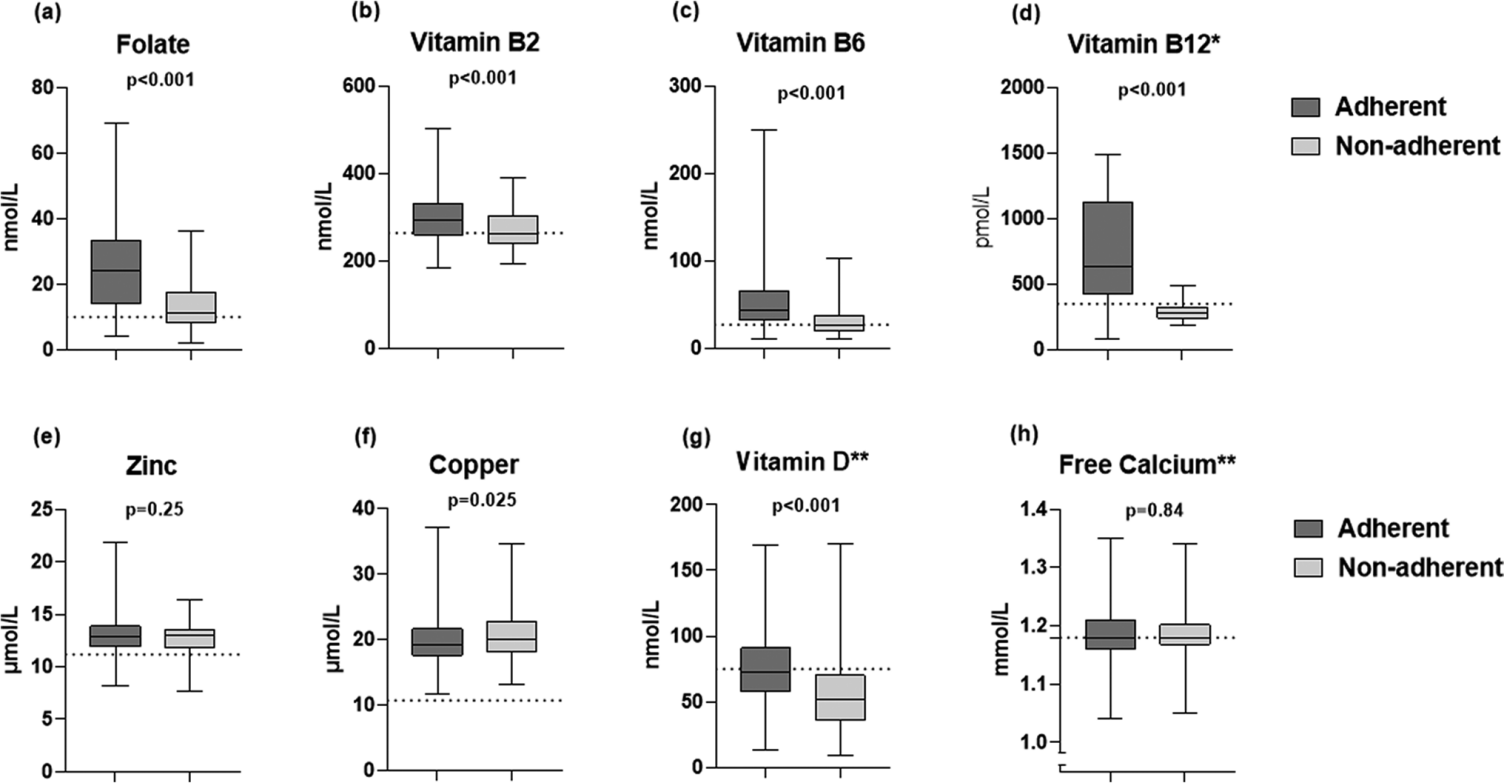
Median (min, max) serum concentrations of folate (**a**), vitamin B_2_ (**b**), vitamin B_6_ (**c**), zinc (**e**), and copper (**f**) in patients adherent or non-adherent to multivitamin-mineral supplements. *Median (min, max) serum concentrations of vitamin B_12_ (**d**) in patients adherent or non-adherent to vitamin B_12_ supplements **Median (min, max) serum concentrations of vitamin D (**g**) and free calcium (**h**) in patients adherent or non-adherent to calcium with vitamin D supplements. Lower reference values are marked with a dotted line

Table 3 presents laboratory test results for the study population 11.7 years after surgery. Vitamin B_2_ (Fig. 2b) and vitamin B_6_ (Fig. 2c) below lower reference value were present in 103 (29%) and 63 (17%) of patient’s adherent, and 67 (52%) and 67 (52%) of patients non-adherent to multivitamin-mineral supplements (*p* < 0.001 in both). Thiamine levels indicating deficiency were present in one (less than 1%) adherent and in six (5%) non-adherent patients (*p* < 0.001). Folate (Fig. 2a) was below the decision limit in 39 (11%) adherent and 44 (34%) non-adherent patients (*p* < 0.001). Folate deficiency with simultaneous high homocysteine was present in 37 (8%) patients, seen more often in patients non-adherent 16/129 (12.4%) vs adherent 21/361 (5.8%) (*p* = 0.016).

**Table 3:** Biochemistry results for patient’s 12 years after Roux-en-Y gastric bypass surgery

Vitamins and minerals (normal range values)	**Adherent Median (IQR)** **n=361**	**Non-adherentMedian (IQR)n=129**	*p*-value	**All Median (IQR)n=490**	**All patients with deficiency n (%)**	**All patients above upper limit of normal range n (%)**
Thiamine (122 nmol/L)	193 (47)	166 (40)	< 0.001	186 (47)	7 (1)	
Vitamin B_2_ (264 nmol/L)	292 (73)	261 (66)	< 0.001	284 (70)	170 (35)	
Vitamin B_6_ (27 nmol/L)	44 (34)	26 (17)	< 0.001	38 (32)	130 (27)	
Folate (> 10 nmol/L*)	24 (20)	11 (10)	< 0.001	19 (20)	83 (17)	
Vitamin A (M: 1.1–2.9 µmol/L)	1.9 (0.7)	1.6 (0.6)	0.037	1.8 (0.6)	5 (5)	13 (13)
Vitamin A (W: 1.2–2.4 µmol/L)	1.8 (0.6)	1.7 (0.5)	0.098	1.8 (0.6)	9 (2)	20 (5)
Zinc (11.2–19.0 µmol/L)	12.9 (2.0)	13.0 (1.7)	0.250	13.0 (1.9)	52 (11)	1 (< 1)
Copper (10.7–22.0 µmol/L)	19.2 (4.2)	20.0 (4.8)	0.024	19.5 (4.2)	0	119 (24)
Vitamin B_12_ ♦ (> 350 pmol/L*)	635 (704)	278 (89)	< 0.001	611 (697)	94 (19)	
Vitamin B_12_ ♦ (> 186 pmol/L)					7/490 (1)	
Vitamin D ♣ (> 75 nmol/L*)	73 (33)	52 (34)	< 0.001	68 (38)	294 (60)	
Free calcium ♣ (1.18–1.32 mmol/L)	1.18 (0.05)	1.18 (0.03)	0.836	1.18 (0.05)	190 (39)	4 (< 1)

Data are presented as median (IQR) and frequencies (percentages) of adherent, non-adherent and all patients

^*^For the vitamin’s folate, vitamin B_12_, and vitamin D, the decision limits are presented instead of reference normal range values

♦Number of patients adherent to vitamin B_12_ 466/490

♣Number of patients adherent to calcium with vitamin D 336/490

*M* Men, *n* = 97; *W* Women, *n* = 393

High vitamin A levels were present in both adherent and non-adherent patients (ns in women, *p* = 0.035 in men). Eight (2%) patients had a vitamin A deficiency, while 25 (5%) had levels above the normal range.

Zinc (Fig. 2e) deficiency was seen in 35 (10%) of the patient’s adherent to multivitamin-mineral supplements and 17 (13%) of the non-adherent patients (*p* = 0.25). A deficiency of copper (Fig. 2f) was not found, but 80 (22%) of the adherent and 39 (30%) non-adherent patients had copper levels above upper reference levels (*p* = 0.025). Among those with high copper levels, six patients had elevated ceruloplasmin levels.

Although adherence to vitamin B_12_ (Fig. 2d) was as high as 466 (95%), sub-optimal levels were seen in 73 (16%) patients, compared to 21 (75%) patients in the non-adherent group (*p* < 0.001). Seven patients had below 186 pmol/L, which was present in both adherent/non-adherent patients.

Adherence to calcium/vitamin D supplements (1000 mg/20 µg) was reported by 336 (69%) patients (Fig. 2g). Sub-optimal level (< 75 nmol/l) of 25-hydroxy vitamin D was seen in 174/336 (52%) adherent patients, compared to 120/154 (78%) non-adherent patients (*p* < 0.001). Parathyroid hormone (PTH) (Table 4) was above the reference level in 196 (40%) patients. Among all patients, 190 (39%) had levels below the normal range for free calcium (Fig. 2h), while four patients (< 1%) had values above (*p* = 0.84).

**Table 4:** Frequency and prevalence of patient’s adherent (A-Ca) or non-adherent (NA-Ca) to Calcium supplement with vitamin D and normal or elevated PTH at different levels of Vitamin D

Vitamin D	A-Ca	NA-Ca	Pearson χ^2^	Normal PTH	Elevated PTH	Pearson χ^2^
25-OH D < 50 nmol/L	50 (10%)	73 (15%)		53 (11%)	70 (14%)	
25-OH D > 50 nmol/L	286 (58%)	81 (17%)	*P* < 0.001	241 (49%)	126 (26%)	*P* < 0.001
25-OH D < 75 nmol/L	174 (36%)	120 (25%)		143 (29%)	151 (31%)	
25-OH D > 75 nmol/L	162 (33%)	34 (7%)	*P* < 0.001	151 (31%)	45 (9%)	*P* < 0.001
25-OH D < 100 nmol/L	278 (57%)	146 (30%)		247 (50%)	177 (36%)	
25-OH D > 100 nmol/L	58 (12%)	8 (2%)	*P* < 0.001	47 (10%)	19 (4%)	*P* = 0.03

The OR of having blood values in the normal or optimal range were 2.7, 5.2, 4.3, and 3.3 times higher in adherent patients vs non-adherent patients for vitamin B_2_, vitamin B_6_, folate, and vitamin D, respectively, all *p*-values < 0.001.

## Discussion

The main finding in our study was that three out of four patients followed these recommendations of life-long multivitamin-mineral supplements 12 years after RYGB. Nearly all patients used recommended vitamin B_12_, and seven out of ten used calcium with vitamin D. The patients used over-the-counter supplements, as specialized multivitamin-mineral supplements for RYGB patients were unavailable until 2017 in Norway. Some patients experience side effects from supplements [11], which might contribute to the lower adherence rate of the chewing calcium supplement in this study. Still, compared to studies from other countries, adherence to recommended supplements in the present study was high [8–10].

Serum folate level has been suggested to indicate adherence to multivitamin-mineral supplementation [6]. Ten percent of the adherent patients and more than 30% of the non-adherent patients were below the decision limit of folate. At the same time, vitamin B_2_ and vitamin B_6_ deficiency were more common in both adherent (30% and almost 20%, respectively) or/and non-adherent (in more than 50%) patients. Another ten-year follow-up study of 431 with RYGB found lower prevalence of folate deficiency (< 8 nmol/L) with 12% of the total patient population while 24% used a multivitamin and 20% used a folate supplement [10]. If folate is low, the patients may be deficient in several B vitamins and the recommendation of multivitamin supplements is reasonable. Some countries fortify food with synthetic folate (folic acid), which is not the case in Norway.

Zinc and copper are absorbed predominantly in the duodenum and jejunum, competing with iron [18]. We found no correlation between zinc and copper and no difference between adherent or non-adherent patients regarding zinc deficiency. Reports on long-term results of copper are scarce, but a two-year follow-up study found 9.6% of the patients to be deficient in copper [19]. We did not find copper deficiency, but one in four had high values of copper. Only six of these also had elevated ceruloplasmin, and the high copper values are probably due to medical conditions. This is not in accordance with a recent pre-and one-year postoperative study, which found a median below the reference level [20].

A deficiency of vitamin B_12_ can result in neurologic complications such as peripheral neuropathy, ataxia, cognitive impairment, depression, and fatigue in mild or extreme forms [21]. One in four patients in BAROBS used per-oral vitamin B_12_ supplements, while the rest of the adherent patients used hydroxocobalamin intramuscular injections every second or third month. The American Society for Metabolic and Bariatric Surgery (ASMBS) Guidelines 2019 recommends orally 350–1000 µg/day or 1000 µg/month intramuscularly or subcutaneously [5]. Vitamin B_12_ is measured as circulating total B_12_, which leaves the biologically active vitamin B_12_ level unknown. Clinically, methylmalonic acid (MMA) and homocysteine are only measured when vitamin B_12_ and folate deficiency are suspected. Almost one in five patients in the present study had elevated MMA, indicating that the supplementation of vitamin B_12_ is too low. It has been suggested that giving vitamin B_12_ injections at patients’ request is harmless, as there is no defined tolerable upper level [22, 23]. The high adherence rate to vitamin B_12_ supplements in the BAROBS study is similar to findings in a five-year study from Norway [7]. Public hospitals and national reimbursement of vitamin B_12_ supplements may contribute to the high adherence rate as well as the symptoms of vitamin B_12_ deficiency are more recognizable for the patients with lack of energy, irritability, and emotional lability. Only seven patients (1.4%), both adherent and non-adherent, had vitamin B_12_ below 186 pmol/L.

Most patients were adherent to recommended calcium with vitamin D supplements, and the multivitamin-mineral supplement also contained vitamin D. Nevertheless, only 40% of the patients had vitamin D above 75 nmol/L, and 40% had free calcium below the reference value. Vitamin D enhances calcium and phosphorus absorption, and low levels may have contributed to low BMD. Ten years after surgery, the most significant fall (25%) in BMD occurred within the first 5 years after surgery [24]. The elevated PTH occurrence in the BAROBS study is comparable to another study with a seven-year follow-up of 70 patients with RYGB and 72 patients with a sleeve gastrectomy [25]. Nine percent (45/490) of the patients in the BAROBS study had elevated PTH levels even when vitamin D was above 75 nmol/L. Recent studies have shown that a vitamin D level above 75 nmol/L is needed to have a PTH in the normal range after RYGB [24, 26, 27]. The BAROBS study supports these findings and even adherent patients need more vitamin D. The BOMSS Guidelines 2020 recommends a higher and more specific dose of vitamin D compared to the Guidelines of 2016; the new range is 50–100 µg/day (2000–4000 IU) until 75 nmol/L is reached [4] [28]. Patients’ adherent are more than three times more likely to have an optimal (> 75 nmol/L) vitamin D level than non-adherent patients.

The main strengths of the BAROBS study are a long-term follow-up (mean 11.7 years), the high number of comparable surgical patients (*n* = 490) and data reflecting a real-time follow-up of patients after obesity surgery. The recommendations for supplements and the follow-up program have been similar in the region. However, the self-reported use of supplements might be overestimated. At time of surgery in 2003–2009 and during the two-to-five-year postoperative follow-up, the patients were recommended an over-the-counter multivitamin-mineral supplement, calcium/vitamin D (1000 mg/20 µg) and vitamin B_12_ injections. Only vitamin B_12_ was reimbursed. Bariatric supplements became available in Norway in 2017 but were more expensive and not easily accessible. It is also considered a limitation that we do not know the daily/weekly number of multivitamin-mineral or calcium/vitamin D tablets the patients took. We assume that most patients followed the recommendations given at the time of surgery; however, some patients may have increased their supplements according to updated guidelines after advice from health professionals, or on their own initiative.

Laboratory tests on vitamins have some limitations on individual levels as the values are snapshots of the patient’s status, and the water-soluble vitamins fluctuate with intake [29]. However, this is not considered a limitation with the high number of patients in the BAROBS study. The BAROBS study may underestimate the general deficiency rate in a post-RYGB population. We may assume that patients (384/930) lost to follow-up may be less adherent to the lifestyle changes and multivitamin-mineral use than patients participating.

Continuous improvement in patient education programs either in-person or digitally with a multidisciplinary team, emphasizing life-long supplementation, the first few years after surgery empowers the patient’s ability to take healthy choices. Updated information on follow-up after obesity surgery should be easily available to general practitioners responsible for the long-term care for these patients. The advice regarding supplements must be easy to follow and the supplements affordable.

## Conclusion

Adherence to supplements, though in sub-optimal doses of new recommendations, decrease the probability of vitamin and mineral deficiency, especially for thiamine, vitamin B_2_, vitamin B_6_, folate, vitamin B_12_, and vitamin D, but does not eliminate it. Twelve years after RYGB, most patients follow the recommendations of daily multivitamin-mineral, vitamin B_12,_ and calcium with vitamin D. These results may be relevant for medical practitioners and multidisciplinary teams treating patients with obesity and after bariatric surgery.

### Supplementary Information

Below is the link to the electronic supplementary material.

Supplementary file1 (DOCX 21 KB)

## Data Availability

The raw data supporting the conclusions of this article will be made available by the authors on request.

## References

[1] Mohapatra S, Gangadharan K, Pitchumoni CS (2020). Malnutrition in obesity before and after bariatric surgery. Dis Mon.

[2] WHO (2017) Nutritional anaemias: tools for effective prevention and control. Report No.: 9789241513067. https://www.who.int/publications/i/item/9789241513067.

[3] Bielawska B, Ouellette-Kuntz H, Patel SV (2020). Severe nutritional complications after bariatric surgery in Ontario adults: a population-based descriptive study. Surg Obes Relat Dis.

[4] O'Kane M, Parretti HM, Pinkney J (2020). British Obesity and Metabolic Surgery Society Guidelines on perioperative and postoperative biochemical monitoring and micronutrient replacement for patients undergoing bariatric surgery-2020 update. Obes Rev.

[5] Mechanick JI, Apovian C, Brethauer S (2020). Clinical practice guidelines for the perioperative nutrition, metabolic, and nonsurgical support of patients undergoing bariatric procedures - 2019 update: cosponsored by american association of clinical endocrinologists/american college of endocrinology, the obesity society, american society for metabolic and bariatric surgery, obesity medicine association, and american society of anesthesiologists. Obesity (Silver Spring).

[6] Auge M, Menahem B, Savey V, et al. Long-term complications after gastric bypass and sleeve gastrectomy: what information to give to patients and practitioners, and why? J Visc Surg. 2022;159(4):298–308. 10.1016/j.jviscsurg.2022.02.004 35304081

[7] Aaseth E, Fagerland MW, Aas AM (2015). Vitamin concentrations 5 years after gastric bypass. Eur J Clin Nutr.

[8] Heusschen L, Berendsen AAM, Cooiman MI (2021). Optimizing multivitamin supplementation for sleeve gastrectomy patients. Obes Surg.

[9] Johansson K, Svensson PA, Söderling J (2021). Long-term risk of anaemia after bariatric surgery: results from the Swedish Obese Subjects study. Lancet Diabetes Endocrinol.

[10] Karefylakis C, Näslund I, Edholm D (2015). Prevalence of anemia and related deficiencies 10 years after gastric bypass–a retrospective study. Obes Surg.

[11] Smelt HJM, Heusschen L, Theel W (2021). Factors affecting patient adherence to multivitamin intake after bariatric surgery: a multicentre survey study from the patient’s perspective. Obes Surg.

[12] Bjerkan KK, Sandvik J, Nymo S, et al. The long-term impact of postoperative educational programs on weight loss after Roux-en-Y gastric bypass. Obes Surg. 2022;32(9):3005–3012. 10.1007/s11695-022-06187-6 PMC9392699 35790673

[13] Olbers T, Lönroth H, Fagevik-Olsén M (2003). Laparoscopic gastric bypass: development of technique, respiratory function, and long-term outcome. Obes Surg.

[14] Sandvik J, Bjerkan KK, Græslie H (2021). Iron deficiency and anemia 10 years after Roux-en-Y gastric bypass for severe obesity. Front Endocrinol (Lausanne).

[15] de Benoist B (2008). Conclusions of a WHO Technical Consultation on folate and vitamin B12 deficiencies. Food Nutr Bull.

[16] Bjørke-Monsen AL, Renstrøm R. What is optimal folate status? Tidsskr Nor Laegeforen. 2020;140(7). https://tidsskriftet.no/en/2022/09/laboratory/what-optimal-folate-status. 10.4045/tidsskr.19.0588 32378856

[17] Bjørke-Monsen AL. Assessment of cobalamin status. Tidsskr Nor Laegeforen. 2020;140(9). https://tidsskriftet.no/en/2022/09/laboratory/assessment-cobalamin-status. 10.4045/tidsskr.19.0589 32549021

[18] Mahawar KK, Bhasker AG, Bindal V (2017). Zinc Deficiency after gastric bypass for morbid obesity: a systematic review. Obes Surg.

[19] Gletsu-Miller N, Broderius M, Frediani JK (2012). Incidence and prevalence of copper deficiency following Roux-en-y gastric bypass surgery. Int J Obes (Lond).

[20] Meyer Mikalsen S, Aaseth J, Flaten TP (2020). Essential trace elements in Norwegian obese patients before and 12 months after Roux-en-Y gastric bypass surgery: copper, manganese, selenium and zinc. J Trace Elem Med Biol.

[21] Lewis CA, de Jersey S, Seymour M (2020). Iron, Vitamin B(12), folate and copper deficiency after bariatric surgery and the impact on anaemia: a systematic review. Obes Surg.

[22] Green R, Allen LH, Bjørke-Monsen AL (2017). Vitamin B(12) deficiency. Nat Rev Dis Primers.

[23] Syn NL, Cummings DE, Wang LZ (2021). Association of metabolic-bariatric surgery with long-term survival in adults with and without diabetes: a one-stage meta-analysis of matched cohort and prospective controlled studies with 174 772 participants. Lancet.

[24] Raoof M, Näslund I, Rask E (2020). Bone mineral density, parathyroid hormone, and vitamin D after gastric bypass surgery: a 10-year longitudinal follow-up. Obes Surg.

[25] Bühler J, Rast S, Beglinger C (2021). Long-term effects of laparoscopic sleeve gastrectomy and Roux-en-Y gastric bypass on body composition and bone mass density. Obes Facts.

[26] Mokhtari Z, Hosseini E, Zaroudi M (2022). The effect of vitamin D supplementation on serum 25-hydroxy vitamin D in the patients undergoing bariatric surgery: a systematic review and meta-analysis of randomized clinical trials. Obes Surg.

[27] Hewitt S, Kristinsson J, Aasheim ET (2020). Relationships between vitamin D status and PTH over 5 years after Roux-en-Y gastric bypass: a longitudinal cohort study. Obes Surg.

[28] O'Kane M, Parretti HM, Hughes CA (2016). Guidelines for the follow-up of patients undergoing bariatric surgery. Clin Obes..

[29] Syn NL, Lee PC, Kovalik JP (2020). Associations of bariatric interventions with micronutrient and endocrine disturbances. JAMA Netw Open.

