# Supplementary material for: Vitamin and Mineral Deficiency 12 Years After Roux-en-Y Gastric Bypass a Cross-Sectional Multicenter Study
Source: Obes Surg. 2023 Aug 27;33(10):3178–85. doi: 10.1007/s11695-023-06787-w (PMC10514116; doi:10.1007/s11695-023-06787-w)
Supplement: Supplementary file 1 — Supplementary file1 (DOCX 21 KB) [file 11695_2023_6787_MOESM1_ESM.docx]

At St. Olavs Hospital the following vitamins and minerals were analyzed with these methods:

Thiamine (B_1)_, vitamin B_2_, vitamin B_6_, vitamin A for all three hospitals using Agilent 1100 with ChemStation software (Agilent Technologies, Matriks, Oslo, Norway). Folate, vitamin B_12_ and PTH were analyzed using Advia Centaur XPT, Siemens Healthineers, Oslo, Norway. Vitamin D (25-OH-vitamin D) were analyzed by Acquity UPLC® I Class with Xevo TQS MSMS (Waters, Oslo, Norway). Zinc and copper from all three hospitals were analyzed by AAnalyst 800 Atomic Absorption Spectrometer, PerkinElmer (Perkinelmer Norway AS, Oslo, Norway). Free calcium was measured on an ABL90blood gas analyzer (Radiometer, Copenhagen, Denmark.

At Namsos Hospital the following vitamins and minerals were analyzed with these methods:

Folate, vitamin B_12_, vitamin D (25-OH-vitamin D) and PTH were analyzed by Alinity ci, (Abbott Diagnostics, Oslo, Norway). Chemiluminescent microparticle immunoassay, CMIA-technology Chemiflex. Free calcium was analyzed using RAPIDPoint 500 (Siemens Healthineers, Oslo, Norway).

At Aalesund Hospital the following vitamins and minerals were analyzed with these methods: Folate, vitamin B_12_, vitamin D (25-OH-vitamin D) and PTH were analyzed using Cobas, (Roche Diagnostics, Oslo, Norway). Electro Chemi Luminescence technology for immunoassay analysis, ECLIA. Free calcium was analyzed using Rapidpoint 500 (Siemens Healthineers, Oslo, Norway) and ABL 800 Flex (Radiometer, Copenhagen, Denmark).
